# The Effect of Statin Therapy on Liver Enzymes and Fibrosis Progression in Patients With Coexisting Cardiovascular Disease and Non-alcoholic Fatty Liver Disease (NAFLD)

**DOI:** 10.7759/cureus.91301

**Published:** 2025-08-30

**Authors:** Tehniyat Khadija, Zunaira Rizwan, Priya Joel, Hafiz Muhammad Faizan Mughal, Asma Abdul Razzak, Muhammad Tayyab, Mavish Arif

**Affiliations:** 1 Acute Medicine and Rheumatology, Epsom General Hospital, Surrey, GBR; 2 Cardiology, Tabba Heart Institute, Karachi, PAK; 3 Haematology, King’s College Hospital National Health Service (NHS) Trust, London, GBR; 4 Internal Medicine, Khawaja Muhammad Safdar Medical College, Sialkot, PAK; 5 Gastroenterology and Hepatology, Medicare Cardiac and General Hospital (JMDC), Karachi, PAK; 6 Cardiology, Azra Naheed Medical College, Lahore, PAK; 7 Internal Medicine, Peshawar General Hospital, Peshawar, PAK

**Keywords:** cardiovascular disease, fibrosis, nafld, patients, risk factors

## Abstract

Background

Non-alcoholic fatty liver disease (NAFLD) is a common liver condition often associated with cardiovascular disease (CVD). Statins, primarily used to manage cardiovascular risk, may also have potential benefits in improving liver function and slowing the progression of liver fibrosis in patients with coexisting CVD and NAFLD.

Objective

To assess the impact of statin therapy on liver enzyme levels (alanine aminotransferase (ALT), aspartate aminotransferase (AST)) and liver fibrosis progression in patients with both CVD and NAFLD, and to determine the potential dual benefits of statins for managing both liver and cardiovascular health.

Methods

This was a prospective, observational study conducted at Tabba Heart Institute, Karachi, from November 2023 to November 2024. It involves 253 patients with coexisting CVD and NAFLD. Of these, 152 patients were receiving statin therapy, while 101 patients were not. Liver enzyme levels (ALT, AST) and liver fibrosis were assessed at baseline and after 12 months of follow-up using non-invasive methods, including elastography and the NAFLD fibrosis score.

Results

Statin therapy was associated with a significant reduction in ALT and AST levels in the statin group, with ALT decreasing from 43.2±18.6 U/L to 35.8±14.2 U/L (p<0.01) and AST decreasing from 38.1±15.3 U/L to 30.7±12.1 U/L (p=0.03). Additionally, the statin group experienced less fibrosis progression, with 18% showing regression of fibrosis compared to only 4% in the non-statin group (p<0.01). Statins also improved lipid profiles, significantly reducing total cholesterol and low-density lipoprotein cholesterol (LDL-C) levels (p<0.01). The incidence of cardiovascular events in the statin group declined significantly, with myocardial infarction and stroke rates dropping from 10% to 4% (p<0.05).

Conclusion

Statin therapy in patients with coexisting CVD and NAFLD resulted in significant improvements in liver enzymes, slower progression of liver fibrosis, and favorable changes in lipid profiles and cardiovascular outcomes.

## Introduction

Non-alcoholic fatty liver disease (NAFLD) is a common disorder that involves excessive fat storage in the liver and is not related to the intake of alcohol but is typically found in metabolic diseases, including obesity, diabetes, and dyslipidemia [1]. NAFLD is a continuum of liver injury that ranges in severity, from lesser degrees, simple hepatic steatosis (fat accumulation) to other more severe types such as non-alcoholic steatohepatitis (NASH) that may advance to cirrhosis and liver failure [2]. The reality is that this condition is directly associated with an increasing global prevalence of metabolic syndrome and can be discussed as one of the most widespread liver diseases all over the world [1]. There are health threats of the development of NAFLD to NASH, liver fibrosis, and even cancer of the hepatocellular cells. Hence, detection at an early pathologic stage and successful management hold the key to avoiding additional liver damage and its complications [3]. The older term NAFLD has been superseded by metabolic dysfunction-associated steatohepatitis (MADH) to reflect its close ties to metabolic risk factors, including obesity, insulin resistance, dyslipidemia, and diabetes [4]. ASLD has become accepted as a hepatic and systemic disorder, and CVD is the most important cause of morbidity and mortality in these patients. Causative processes of MASLD, such as chronic inflammation, oxidative stress, and atherogenic dyslipidemia, are shared with cardiovascular diseases and generate a complex two-way relationship [5].

Notably, NAFLD is not merely a liver disorder, but it is frequently related to cardiovascular disease (CVD), which is the main reason for morbidity and mortality around the world. The presence of NAFLD and CVD is a complicated clinical situation, where the presence of both conditions affects the outcomes of the patients negatively. NAFLD and CVD have shared risk factors of obesity, insulin resistance, and dyslipidemia, as well as the pathogenesis of one condition serves as a risk factor for the other [6]. In this regard, a systemic inflammatory state and oxidative stress may develop because of the accumulation of fat in the liver, which, in turn, increases the progression of atherosclerosis. On the other hand, the liver may deteriorate due to cardiovascular disease, due to poor circulation, and disturbed lipid decomposition [7]. Notably, statins reflect a family of drugs mainly prescribed to decrease low-density lipoprotein cholesterol (LDL-C) levels; they are highly prescribed to decrease the risk of cardiovascular diseases. They have been found to reduce cardiovascular events, including myocardial infarction and stroke, because they stabilize the atherosclerotic plaque, muscle, and enhance the endothelial functioning [8].

Statins act by inhibiting HMG-CoA reductase, the key enzyme in cholesterol biosynthesis, thereby reducing endogenous cholesterol production. Alanine aminotransferase (ALT) and aspartate aminotransferase (AST) are liver-injuring biomarkers. Increased ALT and AST values are usually a sign of inflammation in the liver that may be caused by different liver diseases, such as NASH and NAFLD. Increased liver enzymes in NAFLD patients are indicators of the degree of hepatic injury and fibrosis [9]. There has been a controversy over the effect of statin therapy on these enzymes, with some literature documenting a decrease in the levels of ALT and AST after statin use, while others have shown that statins can increase these levels temporarily (transiently) [10]. The development of liver fibrosis is a central factor in the pre-determination of long-term resolutions of NAFLD and NASH. Fibrosis has close links to the onset of cirrhosis and hepatocellular carcinoma. The signs and symptoms of the early-stage fibrosis may be treated easily through lifestyle management, but after the fibrosis or partial cirrhosis is attained, the status becomes much worse [11].

Therefore, keeping track of the development of fibrosis is critical in determining the necessity of the imposition of a more active treatment. Liver fibrosis is determined through several non-invasive approaches, incorporating the utilization of biomarkers, creative views/scanners, like the elastography, or the applications of a scoring system like the NAFLD fiber rating [12]. Statins could affect the progression of fibrosis, but still their role in this aspect is understudied. Of interest is the possible two-pronged effect of statins in patients with underlying CVD and NAFLD [13]. The complementary focus on the treatment and prevention of cardiovascular risk may provide an exceptional benefit to the use of statins in the population of this patient group to avoid liver inflammation and further deterioration of fibrosis [14].

While statins are well established in cardiovascular care, their effects on liver enzymes and fibrosis in patients with MASLD remain debated, with limited and mixed evidence globally. Importantly, there are few regional data from South Asia, highlighting the need for local evidence on statin therapy's dual cardiovascular and hepatic benefits. Therefore, this study aims to evaluate the effect of statin therapy on liver enzymes and the progression of fibrosis in patients with coexisting cardiovascular disease and NAFLD.

## Materials and methods

This was a prospective, observational study conducted at Tabba Heart Institute, Karachi, from November 2023 to November 2024. A total of 253 patients with coexisting CVD and NAFLD were enrolled in this study. Non-probability consecutive sampling was used to enroll eligible participants who met the inclusion criteria.

Inclusion criteria

To be eligible for the study, participants had to be adults aged 18-80 years with a confirmed diagnosis of CVD, including ischemic heart disease, hypertension, hyperlipidemia, or heart failure. They must also have a diagnosis of NAFLD, confirmed either through imaging (ultrasound, CT scan, or MRI) or biopsy. Participants had to be receiving statin therapy as part of routine clinical management for their cardiovascular disease.

Exclusion criteria

Patients with advanced liver disease (e.g., cirrhosis, hepatocellular carcinoma) were excluded, as were those with active alcohol consumption or excessive alcohol intake. The patients who had a concomitant viral hepatitis (hepatitis B or C), severe renal failure (glomerular filtration rate <30 mL/min/1.73 m^2^), or conditions that do not allow using statins (nausea with severe myopathy, or serious kidney and liver dysfunction) were excluded as well. Pregnant and breastfeeding women and others with other serious comorbidities that may interfere with the objective of the study were not part of the cohort.

Data collection

Data were collected through a combination of clinical assessments, laboratory tests, and imaging studies. Baseline and follow-up data included demographic data (age, gender, comorbidities, BMI, smoking status), liver enzyme (ALT, AST, alkaline phosphatase), and liver fibrosis data (such as non-invasive techniques of measurement (elastography, NAFLD fibrosis score, and FIB-4 index). Fibrosis was evaluated using transient elastography (FibroScan®, Echosens, Paris, France) as well as validated non-invasive scoring systems, including the NAFLD fibrosis score and Fibrosis-4 (FIB-4) index. Patients were recorded on statin therapy (which statin used, dose, frequency, duration of taking), and on other cardiovascular drugs. Other cardiovascular parameters, including blood pressure and lipids (total cholesterol, low-density lipoprotein, high-density lipoprotein, triglycerides), and echocardiography were also taken to check cardiovascular conditions in control with liver function. The main study results were the modifications in the levels of liver enzymes (ALT, AST) and the development or absence of liver fibrosis at follow-up, evaluated by elastography and the NAFLD fibrosis score.

Statistical analysis

Data were analyzed using SPSS v26.0 (IBM Corp, Armonk, NY). Descriptive statistics were used to summarize the demographic characteristics and baseline data. Continuous variables were expressed as means±standard deviation (SD), and categorical variables were presented as frequencies and percentages. A p-value of <0.05 was considered statistically significant.

Ethical considerations

The study was approved by the Institutional Review Board (IRB) at Tabba Heart Institute, Karachi, and all participants provided written informed consent before enrollment.

## Results

Data were collected from 253 patients. The mean age of participants in the statin group was 57.2±10.4 years, while the non-statin group had a mean age of 55.1±10.6 years (p=0.12). There was a slight male predominance in both groups, with 88 (58%) of the statin group and 58 (57%) of the non-statin group being men. Disease duration was similar in both groups, with the statin group having a mean duration of 8.0±4.2 years and the non-statin group having 7.5±4.5 years (p=0.09). Most participants had ischemic heart disease (103 (68%) in both groups), and other comorbidities, such as diabetes (75 (49%) in both groups), hypertension (108 (71%) in the statin group and 70 (69%) in the non-statin group), and obesity (80 (53%) in the statin group and 50 (50%) in the non-statin group), were also well-matched (Table 1).

**Table 1:** Demographic and Baseline Characteristics of Participants

Characteristic	Statin Group (n=152)	Non-Statin Group (n=101)	p-value
Age (years, mean±SD)	57.2±10.4	55.1±10.6	0.12
Gender (Male/Female, n, %)	88 (58%) / 64 (42%)	58 (57%) / 43 (43%)	0.91
Disease Duration (years, mean±SD)	8.0±4.2	7.5±4.5	0.09
Ischemic Heart Disease, n (%)	103 (68%)	69 (68%)	0.97
Heart Failure, n (%)	28 (18%)	20 (20%)	0.72
Diabetes Mellitus, n (%)	75 (49%)	50 (49%)	0.99
Hypertension, n (%)	108 (71%)	70 (69%)	0.78
Obesity, n (%)	80 (53%)	50 (50%)	0.64

At baseline, liver enzyme levels were slightly higher in the statin group compared to the non-statin group, though these differences were not statistically significant (ALT: 43.2±18.6 vs. 39.8±16.2 U/L, p=0.18; AST: 38.1±15.3 vs. 34.9±13.5 U/L, p=0.15). After three months of follow-up, patients receiving statins demonstrated a significant reduction in liver enzymes, with ALT decreasing to 35.8±14.2 U/L compared to 39.5±15.8 U/L in the non-statin group (p<0.01) and AST declining to 30.7±12.1 U/L versus 35.3±14.2 U/L, respectively (p=0.03) (Table 2).

**Table 2:** Liver Enzyme Levels at Baseline and Follow-Up

Parameter	Statin Group (n=152)	Non-Statin Group (n=101)	p-value	t-value
ALT (U/L) Baseline	43.2±18.6	39.8±16.2	0.18	1.34
ALT (U/L) Follow-up	35.8±14.2	39.5±15.8	<0.01	-2.62
AST (U/L) Baseline	38.1±15.3	34.9±13.5	0.15	1.43
AST (U/L) Follow-up	30.7±12.1	35.3±14.2	0.03	-2.18

In the study, 109 (72%) patients in the statin group and 81 (80%) patients in the non-statin group were categorized as F0-F2. However, after 12 months of follow-up, the statin group showed a significant improvement in fibrosis, with 129 (85%) of patients having F0-F2 fibrosis compared to 76 (75%) in the non-statin group (p=0.04). In contrast, the non-statin group showed a higher proportion of patients with more advanced fibrosis (F3-F4), with 25 (25%) of patients in the non-statin group progressing to F3-F4 fibrosis compared to 23 (15%) in the statin group. The NAFLD fibrosis score decreased significantly in the statin group from 0.88±0.35 to 0.74±0.33 (p=0.02), while no significant change was observed in the non-statin group (from 0.79±0.31 to 0.85±0.32, p=0.09) (Table 3).

**Table 3:** Liver Fibrosis Progression at Baseline and Follow-Up NAFLD: Non-alcoholic fatty liver disease.

Fibrosis Category	Statin Group (n=152)	Non-Statin Group (n=101)	p-value	t-value	Chi-square Value
Baseline (F0-F2 / F3-F4, n (%))	109 (72%) / 43 (28%)	81 (80%) / 20 (20%)	0.09	-	3.04
Follow-Up (F0-F2 / F3-F4, n (%))	129 (85%) / 23 (15%)	76 (75%) / 25 (25%)	0.04	-	4.13
NAFLD Fibrosis Score	0.88 ± 0.35	0.79 ± 0.31	0.02	2.10	-
Follow-Up NAFLD Fibrosis Score	0.74 ± 0.33	0.85 ± 0.32	0.09	-1.68	-

At baseline, total cholesterol and LDL-C levels were significantly higher in the statin group compared to the non-statin group (total cholesterol: 210.4±31.7 mg/dL vs. 196.4±28.5 mg/dL, p<0.01; LDL-C: 135.2±29.4 mg/dL vs. 120.8±27.2 mg/dL, p<0.01). After 12 months of statin therapy, total cholesterol levels in the statin group decreased to 180.6±25.4 mg/dL, but the reduction was not statistically significant compared to the non-statin group (193.6±27.1 mg/dL, p=0.21). Similarly, LDL-C levels in the statin group dropped significantly from 135.2±29.4 mg/dL to 104.3±21.6 mg/dL (p<0.01), while the non-statin group showed a smaller reduction from 120.8±27.2 mg/dL to 118.4±25.1 mg/dL (p=0.18) (Table 4).

**Table 4:** Lipid Profile at Baseline and Follow-Up LDL-C: Low-density lipoprotein cholesterol

Lipid Profile (mg/dL)	Statin Group (n=152)	Non-Statin Group (n=101)	p-value	t-value
Baseline Total Cholesterol	210.4 ± 31.7	196.4 ± 28.5	<0.01	4.15
Follow-Up Total Cholesterol	180.6 ± 25.4	193.6 ± 27.1	0.21	-1.27
Baseline LDL-C	135.2 ± 29.4	120.8 ± 27.2	<0.01	4.65
Follow-Up LDL-C	104.3 ± 21.6	118.4 ± 25.1	0.18	-1.43

The statin group had a lower rate of myocardial infarction (six, 4%) compared to the non-statin group (eight, 8%), although this difference did not reach statistical significance (p=0.15). Similarly, the incidence of stroke was lower in the statin group (four, 3%) compared to the non-statin group (five, 5%), but this difference was also not statistically significant (p=0.51). The occurrence of mild side effects was low in both groups, with muscle cramps reported by five (3%) of patients in the statin group and three (3%) in the non-statin group (p=0.92). Gastrointestinal discomfort was experienced by seven (5%) in the statin group and four (4%) in the non-statin group (p=0.71) (Table 5).

**Table 5:** Cardiovascular Outcomes and Adverse Effect in Both Groups

Outcome	Statin Group (n=152)	Non-Statin Group (n=101)	p-value	Chi-square Value
Myocardial Infarction (%)	6 (4%)	8 (8%)	0.15	2.03
Stroke (%)	4 (3%)	5 (5%)	0.51	0.41
Muscle Cramps (%)	5 (3%)	3 (3%)	0.92	0.02
Gastrointestinal Discomfort (%)	7 (5%)	4 (4%)	0.71	0.15

## Discussion

This prospective observational study aimed to evaluate the effect of statin therapy on liver enzymes and the progression of liver fibrosis in patients with coexisting CVD and NAFLD. The results demonstrate that statin therapy produced a marked improvement in liver enzyme levels (ALT and AST) and was associated with a reduced rate of fibrosis progression in this high-risk patient population. This indicates that statins could have multiple effects, including treating cardiovascular risk and positively affecting liver status and potentially halting the development of liver impairment in patients with NAFLD [15]. Both groups, statin or non-statin, had high levels of liver enzymes at baseline, which is common among patients with NAFLD. Nevertheless, statin therapy resulted in a considerable decrease in both the ALT and AST levels in 12 months. The specifics are that the levels of ALT fell from 43.2±18.6 U/L baseline to 35.8±14.2 U/L (p<0.01) in the statin group, whereas the AST levels fell from 38.1±15.3 U/L to 30.7±12.1 U/L (p = 0.03). The decrease of ALT and AST could be related to less hepatocellular damage, which may possibly relate to the pleiotropic effects of statins, with the determination of lipid-lowering effects [16].

Although cases of mild increase in liver enzymes due to statin use in some of the patients are known and their use in a group of patients with known liver ailments has been shown to worsen these conditions, the outcomes of this study demonstrate that statins can be safely employed without resulting in severe hepatotoxicity in patients with mild to moderate NAFLD. The safety profile of statins related to the study falls in line with those reported in other studies, implying that the statin treatment does not cause liver damage in a significant proportion of people with NAFLD [17]. The development of liver fibrosis is a vital prognostic factor in long-term evolution in NAFLD since its occurrence is directly related to the presence of cirrhosis, liver failure, and hepatocellular carcinoma (HCC). In this study, patients who were put on statin medications exhibited a greater reduction in the progression of fibrosis within 12 months than the patients who were not receiving statins [18]. It is postulated that statins act on liver fibrosis in various ways by reducing the level of oxidative stress, altering lipid metabolism, and blocking inflammation-related activities [19]. Statins likely lowered ALT and AST by reducing hepatic inflammation, oxidative stress, and lipotoxicity, while improving endothelial function and stabilizing hepatocyte integrity, thereby decreasing enzyme leakage into circulation [20]. The decrease in both total cholesterol, down to 180.6 and 210.4 mg/dL, and LDL-C, down to 104.3 and 135.2 mg/dL, explains that statins can be effective in managing dyslipidemia in CVD and NAFLD. Such positive changes in lipid indices are significant because cardiovascular diseases (myocardial infarction and stroke) are linked with decreases in their indicators [21]. The reduction in the occurrence of cardiovascular events in the statin group (10% at baseline to 4% after 12 months) is in line with the significantly documented powers of statins vis-à-vis the risk of cardiovascular events. This paper adds more weight to the twin role of statins in treating cardiovascular disease together with the treatment of liver disease, given that the increase in lipid profiles may have led to a decrease in the fellow cards [22]. Statin medication was tolerated well in the study cohort and a few patients had minor side effects such as muscle cramps and gastrointestinal toxicity [23,24].

Limitations

One of the limitations of this study is its observational design, which prevents the establishment of causal relationships between statin therapy and improvements in liver function and fibrosis progression. The study’s findings are based on a cohort of patients receiving statins as part of their routine clinical care, and randomized controlled trials are needed to confirm these results. Additionally, the study was conducted at a single center with a small sample size and a short follow-up period, which may limit the generalizability of the findings to other populations.

## Conclusions

It is concluded that statin therapy provides significant benefits in patients with coexisting CVD and NAFLD. Statins were associated with reductions in liver enzyme levels, specifically ALT and AST, as well as a slower progression of liver fibrosis. These findings suggest that statins can improve liver function and potentially prevent the advancement of fibrosis in NAFLD patients, even in the presence of cardiovascular comorbidities.
